# Prevalence of Isolated Irritable Bowel Syndrome Among Adults in the Kingdom of Bahrain

**DOI:** 10.7759/cureus.56155

**Published:** 2024-03-14

**Authors:** Zahra Alawi, Wadeeah AlMakna, Fatema Hassan, Marwa Faisal, Hawra Matar, Adel S Alsayyad

**Affiliations:** 1 Internal Medicine/Gastroenterology, Mansoura University Hospitals, Mansoura, EGY; 2 Ophthalmology, Salmaniya Medical Complex, Manama, BHR; 3 Psychiatry, Psychiatric Hospital (Bahrain), Manama, BHR; 4 Family and Community Medicine, Arabian Gulf University, Manama, BHR

**Keywords:** rome iv criteria, prevalence, bahrain, irritable bowel syndrome, ibs

## Abstract

Background: Irritable bowel syndrome (IBS) is a very common gastrointestinal disorder encountered in clinical practice. In this study, we estimated the prevalence of isolated IBS and its associated demographic factors among the adult population in the Kingdom of Bahrain.

Methods: A cross-sectional study was conducted targeting adults in Bahrain aged 18 years and above. Individuals with a prior diagnosis of any bowel ailment were excluded. Data was acquired via a self-administered questionnaire. IBS-specific questions were derived from the validated Rome IV diagnostic questionnaire for adults. The scoring methodology inherent to this questionnaire was used for the diagnosis of IBS. The data collection process remained anonymous. Data was compiled using Excel spreadsheets, and the Statistical Package for Social Sciences (SPSS) was employed for analytical purposes. Associations between IBS and demographical or behavioral characteristics were explored using the Chi-square test.

Results: The prevalence of isolated IBS, adopting the Rome IV criteria, was 156 (18.3%) and IBS-M (mixed) type was 40 (38.1%) of these. IBS was predominantly higher among females compared to males (340 vs 235; 22.6% vs 11.9%). The majority of IBS cases (121, 21%) were in the 41-50 age group. A statistically significant association has been demonstrated between IBS and GERD using Pearson’s chi-squared test (p-value = 0.000). Similarly, it was linked to indigestion (p-value = 0.00).

Conclusions: Although the percentage appeared to be significantly higher than the global prevalence of 4% (using Rome IV criteria), our findings were equivalent to the reports conducted in the Middle East region. Integrating holistic patient assessments, including quality of life metrics, along with anxiety, depression, and vitamin D deficiency, will further enhance the understanding of IBS in Bahrain and its impact on the patients and the health services utilization.

## Introduction

Irritable bowel syndrome (IBS) is a very common gastrointestinal disorder encountered in clinical practice [1]. It is characterized by the presence of abdominal pain with altered stool frequency or form [2].

IBS is classified into four subtypes. First is diarrhea-predominant (IBS-D), second is constipation-predominant (IBS-C), third is mixed diarrhea and constipation (IBS-M), and the last one is unclassified (IBS-U) in which symptoms cannot be categorized into one of the previous three subtypes [3].

IBS is suspected in any patient with chronic abdominal pain and altered bowel habits in the context of unidentified organic disorders/diseases. Many criteria were used to diagnose IBS, but the Rome IV criteria by the Rome Foundation were the most used. According to the Rome IV criteria, there should be recurrent abdominal pain at least once per week for the last three months with at least two of the following criteria: associated with defecation, associated with a change in frequency of stools, or associated with a change in the appearance of stool. The criteria should have been met in the last three months, and the symptoms should have begun at least six months before diagnosis [4].

IBS is the most frequent disease diagnosed by gastroenterologists, accounting for 12% of diagnoses in primary care [5]. Vandvik et al. showed that compared to the general population, individuals with IBS visit the doctor two to three times more frequently annually [6]. Although IBS is not a life-threatening disorder, it can significantly affect a patient’s quality of life, which is reflected directly in medical costs and indirect costs related to work absenteeism and productivity [7,8]. According to a study conducted in China, IBS accounts for 3.3% of the total healthcare budget, with an estimated annual cost per patient of CNY18262.84 (USD2933.08) [9].

Despite the established high global prevalence of IBS, its pathophysiology is still unclear. According to various research, some factors are present to some extent in many patients, including genetic predisposition, altered intestinal motility, intestinal hypersensitivity, and psychological distress and its related disorders. However, none of these factors are necessarily present in every case, and various combinations may be encountered [10].

Globally, the prevalence of IBS is estimated to be between 3.8% and 9.2% (using Rome III and Rome IV criteria consequently) with a higher female predominance [11,12]. It affects all age groups; however, people older than 50 years show lower incidence [13]. The prevalence varies globally due to sociodemographic factors, study methods, or diagnostic criteria [14,15]. Another study showed a prevalence of 18.2% among the whole general population of Saudi Arabia [16], while in Lebanon, the prevalence was 20.1% [17]. There has been no previously conducted study about the prevalence of IBS in the Kingdom of Bahrain.

The study aims to estimate the prevalence of IBS and examine its associated demographic factors among the adult population in the Kingdom of Bahrain.

## Materials and methods

Design

A cross-sectional study was conducted, targeting adults in Bahrain aged 18 years and above.

Sample size estimation

The sample size was determined using a standard random sampling formula:

Sample size = $E^2\times\frac{P(1-P)}{D^2}$

where P = anticipated prevalence, which was taken as 0.2; D = precision, set at 0.05; E = Z-score, 1.96 for 95% confidence.

Using this formula, the sample size was calculated as 246. Given the design effect, the target sample size was expanded to 492 participants.

Eligibility criteria

Inclusion

Bahraini adults aged 18 years and above who consented to participate within the study duration were included.

Exclusion

Non-Bahraini individuals, those below 18 years, individuals with a prior diagnosis of any bowel ailment, and those who were unwilling or incapable of participation were excluded.

Data collection instrument

Data was acquired via a self-administered questionnaire utilizing the Rome IV diagnostic parameters for IBS. This included recurrent abdominal discomfort that began at least six months before diagnosis and exhibited two or more of the following symptoms weekly over the past three months: linked to defecation, changes in stool frequency, and changes in stool appearance.

The instrument also gathered sociodemographic data, including gender, age, and nationality. IBS-specific questions were derived from the validated Rome IV diagnostic questionnaire for adults. The scoring methodology inherent to this questionnaire was used for the diagnosis of IBS.

Procedure for data collection

Researchers disseminated the questionnaires through an online platform (WhatsApp groups) in Bahrain and continued data collection for four weeks until the desired sample size was achieved.

Ethical consideration

Within the online questionnaire platform, participants were first introduced with a concise description detailing the study's objectives, followed by a firm commitment to ensuring the strict confidentiality of their submitted data. The study obtained formal authorization from the Ministry of Health (MoH) research committee.

Data management and analysis

The diagnosis of isolated IBS hinged on the Rome IV criteria, with versions available in both English and Arabic. Research members were responsible for data collection and ensured an ample sample volume after data refinement. The data collection process remained anonymous. Each questionnaire was encoded for streamlined data entry and management, with each being attributed a unique identification code.

Data was compiled using Excel spreadsheets, and the Statistical Package for Social Sciences (SPSS) was employed for analytical purposes. Associations between IBS and demographical or behavioral characteristics were explored using the Chi-square test.

## Results

Out of 622 respondents to the online questionnaire, 47 were omitted due to disclosed medical conditions (ulcerative colitis, Crohn's disease, celiac disease, and lactose intolerance), encompassing lower gastrointestinal disorders or other concerning symptoms. Females constituted 59% (340) of the sample, and males represented 40.9%(235). The predominant age group was 18-30 years, comprising approximately 220 (38.4%) respondents, followed by 114 (25.0%) individuals aged 31-40, 121 (21%) in the 41-50 range, 76 (13.2%) in the 51-60 bracket, and a scant 14 (2.4%) respondents aged above 60, marking the smallest segment. The mean age of the participants is 36 years, with a substantial 63% (334) below 40 years, as shown in Table 1.

**Table 1:** Sociodemographic characteristics of the study sample

Variables		Total	Percent
Gender	Male	235	40.90%
	Female	340	59.10%
Age (years)	18–30	220	38.40%
	31–40	114	25.00%
	41–50	121	21.00%
	51–60	76	13.20%
	>60	14	2.40%

**Figure 1:**
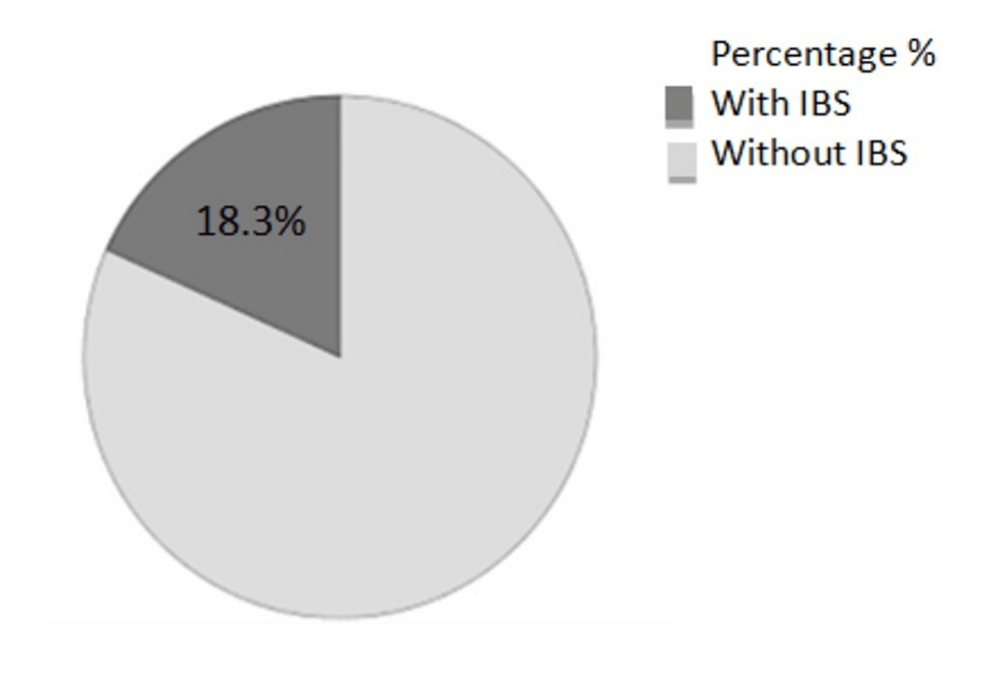
Pie chart representing the prevalence of IBS IBS: Irritable bowel syndrome.

**Figure 2:**
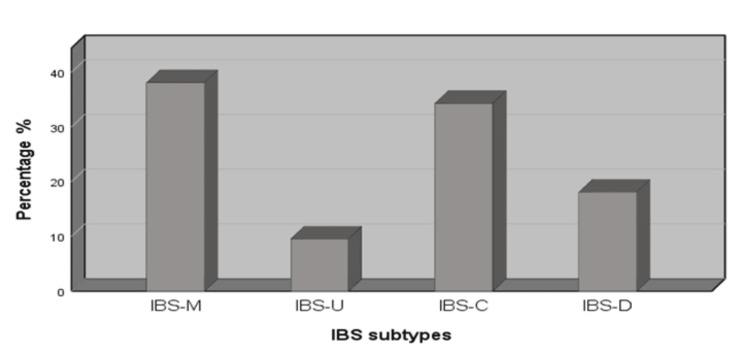
The prevalence of different IBS subtypes IBS: Irritable bowel syndrome; IBS-M: IBS-mixed diarrhea and constipation; IBS-U: IBS-unclassified; IBS-C: IBS-constipation predominant; IBS-D: IBS-diarrhea predominant.

Figure 1 describes that the prevalence of isolated IBS, adopting the Rome IV criteria, was 18.3%, comprising 156 individuals from the overall sample size. The most common subtype was IBS-M 40 (38.1%). Consequently, IBS-C 36 (34.3%), IBS-D 19 (18.1%), and the least prevalent was IBS-U 10 (9.5%). Figure 2 displays the prevalence of different IBS subtypes.

The prevalence of self-reported GERD cases was 101 (16.2%), with 38 (6.10%) cases indicating having peptic ulcer, six (1%) documenting celiac disease, 20 (3.2%) experiencing lactose intolerance, 24 (3.9%) having ulcerative colitis, and four (0.6%) having Crohn’s disease, as shown in Table 2.

**Table 2:** The prevalence of self-reported GIT disorders N: Number; %: Percent; GERD: Gastroesophageal reflux disease; GIT: Gastrointestinal tract.

Disease	N	(%)
GERD	101	16.2%
Peptic ulcer	38	6.1%
Celiac disease	6	1%
Lactose intolerance	20	3.2%
Ulcerative colitis	24	3.9%
Crohn’s disease	4	0.6%

IBS sufferers were asked about the percentage of times they experienced various symptoms (range between 0% and 100%). The average percentage reported by patients for abdominal pain associated with bowel movements was 60.01% of the time. The average percentage of patients experiencing softened or hardened stool coinciding with pain episodes was 66.19% of the time. Changes in stool frequency accompanying pain were reported at an average percentage of 63.3% of the time. Post-meal pain onset or intensification was reported around 52.29% of the time, and disruptions in daily activities were experienced approximately 48.57% of the time, as detailed in Table 3.

**Table 3:** Mean percentage of the population that responded positively to questions relevant to diagnose IBS IBS: Irritable bowel syndrome.

Question	Mean of the percentages
How often did this pain in your abdomen happen close in time to a bowel movement - just before, during, or soon after? (Percent of times with pain)	60.01%
How often did your stools become either softer than usual or harder than usual when you had this pain? (Percent of times with pain)	66.19%
How often did your stools become either more frequent than usual or less frequent than usual when you had this pain? (Percent of times with pain)	63.33%
How often did your pain start or get worse after eating a meal? (Percent of times with pain)	52.29%
When you had this pain, how often did it limit or restrict your usual activities (for example, work, household activities, and social events)? (Percent of times with pain)	48.57%

Isolated IBS was predominantly higher among females (77, 22.6%) than in males (28, 11.9%). The majority of isolated IBS cases (30, 24.8%) were in the 41-50 age group, 31 (21.5%) were in the 31-40 age group, 38 (17.3%) in the 18-30 age group, and 6 (7.9%) in 51-60 age groups. There were no reported cases in the age group above 60 years.

A statistically significant association was demonstrated between IBS and GERD using Pearson’s chi-squared test (p-value = 0.000). Similarly, it has been found to be linked to indigestion (p-value = 0.00). Nevertheless, IBS and peptic ulcer are not significantly associated, as explained in Table 4.

**Table 4:** Summary of IBS prevalence along with overlap with other diseases * Significant associations (p-value < 0.05) n: Number; %: Percent; GERD: Gastroesophageal reflux; IBS: Irritable bowel syndrome.

Variables		Irritable bowel syndrome (IBS) status				P-value
		No		Yes		
		n	(%)	n	(%)	
Gender	Male	207	88.1%	28	11.9%	0.001*
	Female	263	77.4%	77	22.6%	
Age (years)	18–30	182	82.7%	38	17.3%	0.010*
	31–40	113	78.5%	31	21.5%	
	41–50	91	75.2%	30	24.8%	
	51–60	70	92.1%	6	7.9%	
	>60	14	100.0%	0	0.0%	
GERD	Yes	53	63.9%	30	36.1%	0.000*
	No	417	84.8%	75	15.2%	
Peptic ulcer	Yes	25	73.5%	9	26.5%	0.201
	No	445	82.3%	96	17.7%	
Indigestion	Yes	14	56.0%	11	44.0%	0.002*
	No	456	82.9%	94	17.1%	

## Discussion

This current study aimed to investigate the prevalence of isolated IBS in Bahrain using (Rome IV criteria). The results we obtained show that the prevalence was 18.3% (156). In 2019, a cross-sectional study that included both Saudi and Bahraini populations estimated the prevalence to be less than 5% using the Rome II criteria [18]. As far as we know, this is the first review to determine the overall prevalence of IBS among the general population in Bahrain exclusively.

Although the percentage appeared to be significantly higher than the global prevalence of 3.8% (using Rome IV criteria) [11], our findings were equivalent to the reports conducted in the Middle East region. For instance, in Saudi Arabia, the prevalence ranged between 8.9%-40.7% and 31.3% [19]. A cross-sectional study on university students in Syria revealed a prevalence of 17% (using Rome III criteria) [20]. In contrast, a study in Egypt indicated that the prevalence of IBS was found to be 7.6% [11]. Further, some countries like Iran reported a much smaller proportion of 1.1% [7]. The differences across several types of research may be influenced by the diverse data collection methods employed, taking into account other cultural and regional differences [4,21].

Rome III criteria demonstrated a higher prevalence rate compared to studies utilizing the Rome IV criteria. This difference is likely attributed to modifications made in the diagnostic criteria for IBS. The replacement of the term "abdominal discomfort" with "pain" consequently led to a decrease in the reported rate of IBS [22].

According to our study, the most common subtype was IBS-M, accounting for 38.1% (40) of cases, along with IBS-C representing 34.3% (36) of cases. These outcomes were consistent with many prior studies in Saudi Arabia [13,16,23].

In this study, IBS was more prevalent in women than men, which is supported by various reported literature. Nevertheless, some studies conducted in other Asian countries have found no significant gender disparities in the prevalence of IBS [24].

The majority of IBS cases reported were in the 41-50 age group. This contradicts the evidence of some studies, including a research from Jazan, Saudi Arabia, that demonstrated no correlation between age groups and the prevalence of IBS [25].

We have found a statistically significant correlation between IBS and GERD. Several research shows that this overlap is a common occurrence, leading to a substantially higher prevalence of IBS in patients with GERD [24,26,27]. A study conducted in Iran of 6476 IBS patients revealed that 2658 patients were diagnosed with GERD based on clinical findings and endoscopic results representing 63.6% of subjects [20]. Similarly, IBS has also been discovered to be connected to indigestion.

The information utilized in this study was obtained through an online-based self-filled questionnaire, a subjective approach that can potentially introduce bias and limitations in terms of individuals with a lack of internet access or those who are unable to read. However, in a global study, an online-based survey yielded more effective prevalence rate estimates than the traditional epidemiological survey due to the online survey's ability to achieve comprehensive, precise, and dependable data collection [28].

The development of IBS is susceptible to influence from various factors, encompassing the individual's living environment, socioeconomic status, level of education, mental well-being, and dietary habits [29,30]. Thus, an additional limitation of the study is the absence of personal history information about the participants. In addition, the assessment of quality of life (QoL) was not conducted in our study. Further investigations about anxiety, depression, and vitamin D deficiency should be explored alongside IBS. This could facilitate the evaluation of how IBS affects daily functioning from a behavioral standpoint. Further discussion will be required to justify this.

Management strategies in the region mainly focus on dietary changes and home remedies [31] along with psychological interventions [32] and antidepressants [33,34].

## Conclusions

For future research on IBS in Bahrain, it is important to consider the differences in Rome criteria versions and their impact on diagnostic rates. Furthermore, there is a need to focus on potential gender and age-related patterns while also exploring the evident link between IBS and GERD. Integrating holistic patient assessments, including QoL metrics, will further enhance the understanding of IBS in Bahrain and its impact on both patients and health services utilization.

## Figures and Tables

**Figure 3:**
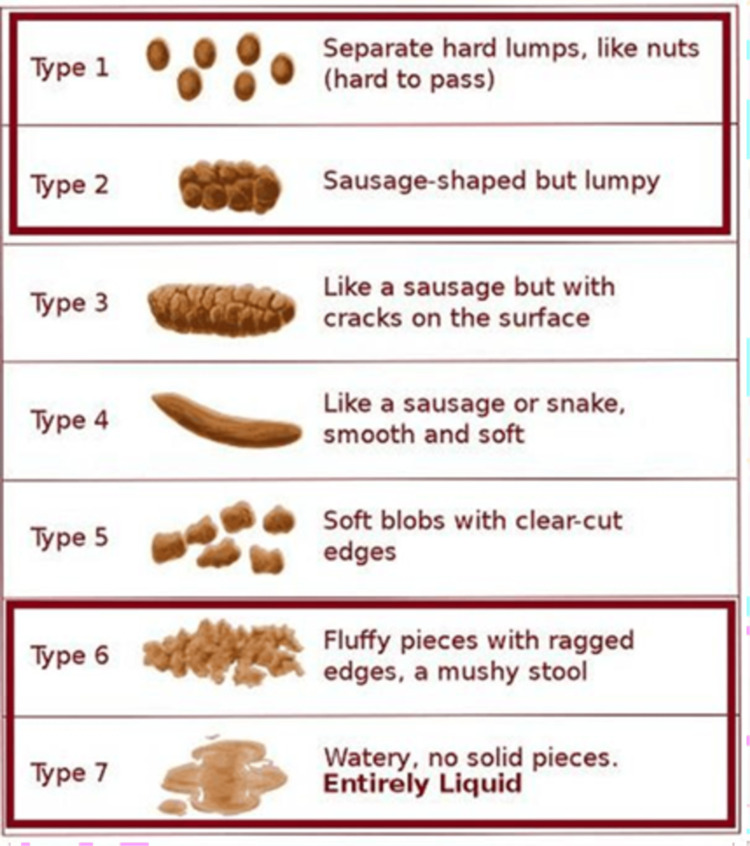
Bowel movement type

**Figure 4:**
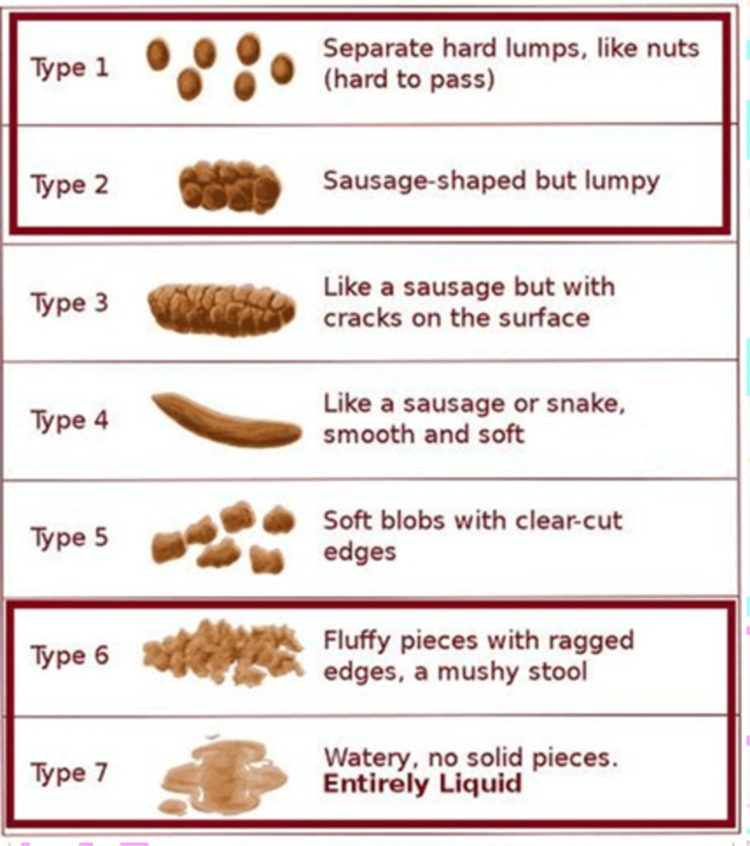
صورة تابعة للسؤال رقم 16

## Appendices

Rome IV diagnostic questionnaire for adults

Hello, we are conducting a study on the prevalence of IBS in Bahrain. We would like to know whether you have signs and symptoms of IBS. Please help us by completing this 20-minute survey. Your responses will be completely anonymous. Thank you for your participation.

1. I agree to participate

- Yes

- No

2. What is your nationality?

- Bahraini

- Non-Bahraini

3. What is your age?

4. What is your gender?

- Male

- Female

5. Have you been diagnosed with any chronic disease related to the gastrointestinal tract?

- Yes

- No

6. If you answered yes to the previous question, please specify.

- GERD

- Indigestion

- Peptic ulcer

- Celiac disease

- Lactose intolerance 

- Crohn’s disease

- Ulcerative colitis

- Others

7. In the last 3 months, how often did you have pain anywhere in your abdomen?

- Never

- Less than one day a month

- One day a month

- Two to three days a month

- Once a week

- Two to three days a week

- Most days

- Every day

- Multiple times per day or all the time

8. How often did this pain in your abdomen happen close in time to a bowel movement - just before, during, or soon after? (Percent of times with pain)

- 0% Never

- 10%

- 20%

- 30%

- 40%

- 50%

- 60%

- 70%

- 80%

- 90%

- 100% Always

9. How often did your stools become either softer than usual or harder than usual when you had this pain? (Percent of times with pain)

- 0% Never

- 10%

- 20%

- 30%

- 40%

- 50%

- 60%

- 70%

- 80%

- 90%

- 100% Always

10. How often did your stools become either more frequent than usual or less frequent than usual when you had this pain? (Percent of times with pain)

- 0% Never

- 10%

- 20%

- 30%

- 40%

- 50%

- 60%

- 70%

- 80%

- 90%

- 100% Always

11. For women: How often did your pain get worse with menstrual bleeding? (Percent of times with pain)

- 0% Never

- 10%

- 20%

- 30%

- 40%

- 50%

- 60%

- 70%

- 80%

- 90%

- 100% Always

12. How often did your pain start or get worse after eating a meal? (Percent of times with pain)

- 0% Never

- 10%

- 20%

- 30%

- 40%

- 50%

- 60%

- 70%

- 80%

- 90%

- 100% Always

13. When you had this pain, how often did it limit or restrict your usual activities (for example, work, household activities, and social events)? (Percent of times with pain)

- 0% Never

- 10%

- 20%

- 30%

- 40%

- 50%

- 60%

- 70%

- 80%

- 90%

- 100% Always

14. Has this pain in your abdomen been continuous or almost continuous? (Continuous means that it never goes away during waking hours)

- No

- Yes

15. Has it been 6 months or longer since you started having this pain?

- No

- Yes

Bowel movements of type 1 or 2 and type 6 or 7 in the figure below can be considered abnormal. Type 1 or 2 means you are constipated, and type 6 or 7 means you have diarrhea.

16. In the last three months, when you had abnormal stools, what were they usually like?

- Usually constipation (like type 1 or 2 in the picture)

- Usually diarrhea (like type 6 or 7)

- Both diarrhea and constipation - that is, more than ¼ of all the abnormal bowel movements were constipation and more than ¼ were diarrhea

- Not applicable, because I never or rarely had abnormal bowel movements

17. In the last 3 months, have you had any of the following symptoms? Check all that apply:

- Red blood in your stools two or more times

- Black stools two or more times

- Vomited blood two or more times

- Temperature over 990 Fahrenheit (380 Centigrade) two or more times

- Unintentionally lost over 10 pounds (4.5 kilograms)

- Major change in bowel movements (change in frequency or consistency)

18. Have you been told by your doctor that you are anemic (a low blood count or low iron)? (If female, not due to your menstrual period)?

- No

- Yes

19. Do you have a parent, brother, or sister who has (or had) one or more of the following? Check all that apply:

- Cancer of the esophagus, stomach, or colon

- Ulcerative colitis or Crohn’s disease

- Celiac disease

Rome IV diagnostic questionnaire for adults, Arabic version

1. ​أوافق على ملئ الاستبيان

نعم -

لا -

2. ​الجنسية

 بحريني -

 غير بحريني-

3. ​العمر

اقل من 18 سنة-

18-30 سنه-

31-45 سنة-

اكبر من 45 سنة- 

4. الجنس؟

ذكر-

أنثى-

5. هل سبق و تم تشخيصك بمرض في الجهاز الهضمي؟

 نعم-

 لا-

6. اذا أجبت بنعم في السؤال رقم 3, هل يمكنك تحديده؟

7. في آخر 3 أشهر، كم مرة عانيت من ألم في أي مكان من بطنك؟

أبدًا-

أقل من يوم واحد في الشهر-

يوم واحد في الشهر-

من يومين إلى ثلاثة أيام في الشهر-

مرة واحدة في الأسبوع-

من يومين إلى ثلاثة أيام في الأسبوع-

أغلب الأيام (من أربعة إلى ستة أيام بالأسبوع)-

كل يوم-

 عدة مرات في اليوم الواحد أو طوال الوقت-

8. كم مرة أصابك هذا الألم في بطنك في وقت قريب من التغوّط؛ إما قبله مباشرةً أو خلاله أو بعده بقليل؟ (النسبة المئوية للمرات المصحوبة بالألم(

 أبدًا-

10%-

20%-

30%-

40%-

50%-

60%-

70%-

80%-

90%-

100% دائمًا-

9. كم مرة أصبح برازك إما أكثر ليونة من المعتاد أو أكثر صلابة من المعتاد عندما أصابك هذا الألم؟ (النسبة المئوية للمرات المصحوبة بالألم)

أبدًا-

10%-

20%-

30%-

40%-

50%-

60%-

70%-

80%-

90%-

100% دائمًا-

10. كم مرة أصبح إخراج البراز إما أكثر تكرارًا من المعتاد أو أقل تكرارًا من المعتاد عندما أصابك هذا الألم؟ (النسبة المئوية للمرات المصحوبة بالألم)

أبدًا-

10%-

20%-

30%-

40%-

50%-

60%-

70%-

80%-

90%-

100% دائمًا-

11. للنساء: كم مرة اشتد فيها ألمكِ مع نزيف الحيض؟ (النسبة المئوية للمرات المصحوبة بالألم)

أبدًا-

10%-

20%-

30%-

40%-

50%-

60%-

70%-

80%-

90%-

100% دائمًا-

لا ينطبق هذا السؤال عليّ-

12. كم مرة بدأ ألمك أو اشتدّ بعد تناول وجبة طعام؟ (النسبة المئوية للمرات المصحوبة بالألم)

أبدًا-

10%-

20%-

30%-

40%-

50%-

60%-

70%-

80%-

90%-

100% دائمًا-

لا ينطبق هذا السؤال عليّ-

13. عندما أصابك هذا الألم، كم مرة قيَّد أو حدَّ من أنشطتك المعتادة (على سبيل المثال، العمل والأنشطة المنزلية والمناسبات الاجتماعية)؟ (النسبة المئوية للمرات المصحوبة بالألم)

أبدًا-

10%-

20%-

30%-

40%-

50%-

60%-

70%-

80%-

90%-

100% دائمًا-

لا ينطبق هذا السؤال عليّ-

14. هل كان هذا الألم في بطنك مستمرًا أو تقريبًا مستمر؟ (تعنى مستمر أنه لا يتلاشى إطلاقًا خلال ساعات اليقظة)

لا-

نعم-

15. هل مضت 6 أشهر أو أكثر منذ بدأ يصيبك هذا الألم?

لا-

نعم-

التغوّط من النوع 1 أو 2 وأيضًا من النوع 6 أو 7 في الصورة أدناه يمكن اعتباره غير طبيعي. النوع 1 أو 2 يعني أنك مصاب بالإمساك، والنوع 6 أو 7 يعني أنك تعاني من الإسهال.

16. في آخر 3 أشهر، عندما كان البراز غير طبيعى، ماذا كان يشبه عادةً؟

عادةً إمساك (مثل النوع 1 أو 2 في الصورة)-

عادةً إسهال (مثل النوع 6 أو 7)-

كلاً من الإسهال والإمساك - وهذا يعني، أكثر من 1/4 كل مرات التغوطات غير الطبيعية كانت إمساك وأكثر من الـ 1/4 كان إسهال-

غير منطبق، لأني أبدًا أو نادرًا ما كان عندي تغوطات غير طبيعية-

17. في آخر 3 أشهر، هل عانيت من أي من الأعراض التالية؟ ضع علامة على كل ما ينطبق:

دم أحمر في برازك مرتين أو أكثر؟-

براز أسود مرتين أو أكثر؟-

تقيأت دمًا مرتين أو أكثر؟-

رجة الحرارة أعلى من 99 درجة فهرنهايت (38 درجة مئوية) مرتين أو أكثر-

خسرت من وزنك أكثر من 10 أرطال (4.5 كجم) عن غير قصد؟-

تغيّر كبير في التغوّط (تغيّر في عدد مرات التغوُّط أو شكل البراز)؟-

18. هل سبق أن أخبرك طبيبك أنك مصاب بفقر الدم (لديك نقص فى تعداد خلايا الدم أو نقص في الحديد)؟

لا-

نعم-

19. هل لديك أب أو أم أو أخ أو أخت مصاب (أو كان مصابًا) بواحد أو أكثر مما يلي؟ ضع علامة على كل ما ينطبق:

سرطان المريء أو المعدة أو القولون؟-

داء كرون أو التهاب القولون التقرّحي ؟-

مرض السيلياك (حساسية الإمعاء للقمح)؟-

Scoring algorithm for the Rome IV diagnostic questionnaire for adults

Irritable bowel syndrome must fulfil the following criteria for the past three months:

1. Recurrent abdominal pain (Q7 = at least weekly)

2. Pain is associated with two or more of the following criteria:

- Related to defecation (Q8 = at least 30% of occasions)

- Associated with a change in frequency of stool (Q9 = at least 30% of occasions)

- Associated with a change in form (appearance) of stool (Q10 = at least 30% of occasions)

3. Symptom onset at least six months before diagnosis (Q15 = yes)

When using the Rome IV diagnostic questionnaire, IBS subtypes are based on the patient's perception of the usual consistency of abnormal stools. Question 16 uses a picture of the Bristol Stool Scale to define abnormal stools and to classify them as follows:

- IBS-C if abnormal stools are usually constipation (types 1-2)

- IBS-D if abnormal stools are usually diarrhea (types 6-7)

- IBS-M if abnormal stools are mixed with at least 1/4 constipation AND at least 1/4 diarrhea

- IBS-U if the subject never or rarely has abnormal stools
